# Sepiapterin Enables Liberalisation of Natural Protein Intake While Improving Metabolic Control in Adolescents with Phenylketonuria

**DOI:** 10.3390/nu18203376

**Published:** 2026-10-15

**Authors:** Alex Pinto, Anne Daly, Suresh Vijay, Sharon Evans, Anita MacDonald

**Affiliations:** Birmingham Children’s Hospital, Steelhouse Lane, Birmingham B4 6NH, UK; a.daly3@nhs.net (A.D.); suresh.vijay1@nhs.net (S.V.); sharon.morris6@nhs.net (S.E.); anita.macdonald@nhs.net (A.M.)

**Keywords:** sepiapterin, phenylketonuria, phenylalanine, natural protein intake, metabolic control

## Abstract

**Background:** Sepiapterin is a synthetic precursor of tetrahydrobiopterin (BH_4_) that increases intracellular BH_4_ availability and has demonstrated greater phenylalanine-lowering efficacy than sapropterin. We evaluated its effect on dietary liberalisation and metabolic control in adolescents with phenylketonuria (PKU). **Methods:** Seven adolescents with PKU (median age of commencing sepiapterin: 13 years, range 12–17 years), including five previously responsive to sapropterin, were studied. Median natural protein intake, protein equivalent from protein substitute, and total protein intake were compared during diet-only treatment, sapropterin treatment (where applicable), and after 12 months of sepiapterin. Blood phenylalanine (Phe) and tyrosine (Tyr) concentrations were assessed over 12 months for each treatment period. **Results:** Median natural protein intake increased from 6.5 g/day (range 4.5–15) during diet-only treatment (n = 7) to 20 g/day (10–25) with sapropterin (n = 5) and 70 g/day (34–90) after 12 months of sepiapterin (n = 7). This represented a median increase of 218% (70–471%) compared with sapropterin and 600% (200–1900%) compared with diet alone. Median total protein intake on sepiapterin was 90 g/day (70–114), equivalent to 1.4 g/kg/day, and four participants discontinued protein substitute completely. Mean (±SD) blood Phe concentrations were lower with reduced variability on sepiapterin (303 ± 157 µmol/L during diet-only treatment; 321 ± 136 µmol/L with sapropterin; 260 ± 116 µmol/L with sepiapterin), while Tyr concentrations remained stable. The percentage of blood Phe concentrations within target improved with sepiapterin (≤360 µmol/L: 83%, range 77–100) compared with diet alone (68%, range 17–100) and sapropterin (66%, range 40–91). Most participants incorporated high-protein foods into their diets, including meat (7/7), fish (6/7), cheese (6/7), and milk or yoghurt (5/7). **Conclusions:** Twelve months of sepiapterin enabled marked dietary liberalisation while improving metabolic control in adolescents with PKU. These findings suggest that sepiapterin may substantially reduce treatment burden and represent an important advance in the management of this challenging age group.

## 1. Introduction

Despite universal newborn screening and early initiation of treatment, adolescence remains one of the most challenging periods for maintaining metabolic control in phenylketonuria (PKU) [1,2,3]. Blood phenylalanine (Phe) concentrations commonly increase during adolescence as greater autonomy, ongoing maturation of executive function, academic demands, and a desire for social integration compromise adherence to treatment [4,5]. Sustained elevations in blood Phe during this critical stage are associated with impairments in attention, executive function, processing speed, mental health, and educational achievement, with adverse effects that may persist into adulthood [6,7].

Primary management of PKU is dietary treatment [8,9]. Restriction of natural protein, supplemented with low/Phe-free protein substitutes and specialised low-protein foods, effectively prevents the neurotoxic consequences of hyperphenylalaninaemia when metabolic control is maintained [10,11,12,13,14]. However, this regimen imposes a substantial lifelong treatment burden [15,16], requiring meticulous dietary planning, frequent blood Phe monitoring, and sustained adherence. During adolescence, responsibility for treatment progressively transfers from caregivers to the individual, while school, sport, social activities and peer influences increasingly compete with treatment priorities [17,18]. Consequently, many adolescents fail to achieve recommended blood Phe targets despite specialist multidisciplinary care [19,20,21].

Pharmacological therapies have broadened treatment options for phenylketonuria (PKU), yet significant therapeutic limitations remain. Sapropterin, a synthetic formulation of tetrahydrobiopterin (BH4), improves metabolic control in individuals with BH4-responsive PKU; however, responsiveness is confined to approximately 30–40% of patients, and increases in natural protein tolerance (defined as the maximum natural protein intake that can be achieved while maintaining blood Phe concentrations within the target range) are often modest despite biochemical improvement [20,21]. Furthermore, the pharmacokinetic properties of sapropterin limit its therapeutic efficacy. Following oral administration, BH4 undergoes rapid oxidation to dihydrobiopterin (BH2) within the gastrointestinal tract and circulation and is extensively cleared by renal organic anion transporters, resulting in low bioavailability and short systemic exposure [22,23]. Consequently, PAH activation may be incomplete, leaving many adolescents dependent on intensive dietary restriction, low-Phe protein substitutes, and frequent blood Phe monitoring. These limitations highlight the need for therapies capable of delivering more sustained PAH activation, greater reductions in blood Phe concentrations, and clinically meaningful increases in natural protein tolerance while reducing lifelong treatment burden.

More recently, sepiapterin, a novel oral pharmacotherapy, has been approved in the European Union, the United States, Australia, Switzerland, Canada and Japan. Unlike sapropterin, sepiapterin has a distinct dual mechanism of action that acts both as a pharmacological chaperone that stabilises phenylalanine hydroxylase (PAH) and as a precursor of tetrahydrobiopterin (BH4) through the endogenous pterin salvage pathway, thereby increasing intracellular BH4 availability and enhancing residual enzyme activity [24,25,26].

In the phase III placebo-controlled APHENITY programme, sepiapterin demonstrated superior metabolic efficacy, with approximately 65% of treated participants achieving blood Phe concentrations ≤ 360 μmol/L, alongside clinically important increases in natural protein tolerance and a favourable safety profile [27,28]. In the Phase 3 AMPLIPHY trial, participants who were responsive to sepiapterin underwent a randomized crossover comparison of sepiapterin (60 mg/kg/day; licensed dose) and sapropterin (20 mg/kg/day; maximum licensed dose), with each treatment administered for 4 weeks. Sepiapterin produced a significantly greater reduction in blood Phe concentrations than sapropterin, with a relative 70% greater reduction from baseline (least-squares mean difference −180.4 μmol/L; 95% CI −229.5 to −131.4; *p* < 0.0001) [29].

These findings suggest that sepiapterin has the potential to substantially reduce dietary treatment burden, particularly in adolescents, although evidence in this age group remains limited.

In this case series, we describe the longitudinal dietary, metabolic, and clinical outcomes of adolescents with PKU treated with sepiapterin at a single metabolic centre. We evaluated changes in blood Phe concentrations, natural protein tolerance, protein substitute requirements, and overall protein intake during the first year of treatment with sepiapterin to provide early evidence of its effectiveness in routine clinical practice to complement clinical trial findings.

## 2. Materials and Methods

### 2.1. Study Design and Data Collection

This longitudinal, retrospective case-record review evaluated the 12-month clinical outcomes of adolescents with PKU who had previously participated in the Phase 3 AMPLIPHY and/or APHENITY sepiapterin clinical programmes [27,28,29] and subsequently continued sepiapterin treatment at Birmingham Children’s Hospital. The participants and some of their clinical trial data therefore contributed to these programmes and do not represent an independent cohort. The present analysis addresses distinct questions that were not the primary focus of the Phase 3 reports: it examines an adolescent-specific cohort, provides longitudinal within-patient comparisons with historical periods of dietary treatment alone and, where applicable, sapropterin treatment, and describes in detail the progressive dietary changes during the first 12 months of sepiapterin treatment in routine clinical practice.

Clinical data were collected, where available, across three treatment periods: dietary management alone, sapropterin treatment, and sepiapterin treatment. Diet-alone data were obtained immediately before the sapropterin response trial or, in participants who were non-responsive to sapropterin, immediately before the sepiapterin response trial. For adolescents who responded to sapropterin, dietary data during sapropterin treatment were collected once each participant had achieved their maximum tolerated natural protein intake and before commencing the sepiapterin response trial.

Metabolic control was assessed using blood Phe and tyrosine (Tyr) concentrations (μmol/L) measured from dried blood spots during each treatment period. For the diet-only and sapropterin treatment periods, blood Phe and Tyr data were obtained from routine monitoring records for the 12 months immediately preceding initiation of the subsequent drug treatment. For sepiapterin, blood Phe and Tyr concentrations were assessed during the first 12 months of treatment. Growth outcomes were assessed using measurements recorded at the end of each treatment period and included weight (kg and z-score), height (cm and z-score), and body mass index (BMI; kg/m^2^ and z-score).

Dietary intake was assessed using 24-h dietary recalls during diet-only and sapropterin treatment and 3-day dietary records during the 12-month sepiapterin follow-up. Natural protein intake was calculated from protein-containing foods and expressed as g/day and g/kg/day. Dietary Phe intake was recorded in mg/day, and protein equivalent supplied by protein substitutes was recorded in g/day and g/kg/day. Total protein intake was calculated as natural protein plus protein equivalent from protein substitutes. Natural protein tolerance was defined as the maximum amount of natural protein that could be consumed while maintaining blood Phe concentrations within the clinically recommended target range.

An algorithm was used as part of the Aphenity/Ampliphy trial [27,28,29] to adjust dietary Phe intake according to blood Phe levels:

- Blood Phe 0–180 µmol/L: increase Phe intake by 15 mg/kg/day
- Blood Phe 181–240 µmol/L: increase Phe intake by 10 mg/kg/day
- Blood Phe 241–300 µmol/L: increase Phe intake by 5 mg/kg/day
- Blood Phe 301–359 µmol/L: no change to Phe intake
- Blood Phe ≥360 µmol/L: initially no change to Phe intake but if Phe levels remain elevated, progressively reverse the most recent dietary Phe increases.

If Phe/protein intake reached ≥2 times the recommended daily amount, further increases could be stopped without being considered a protocol deviation.

**Figure 1:**
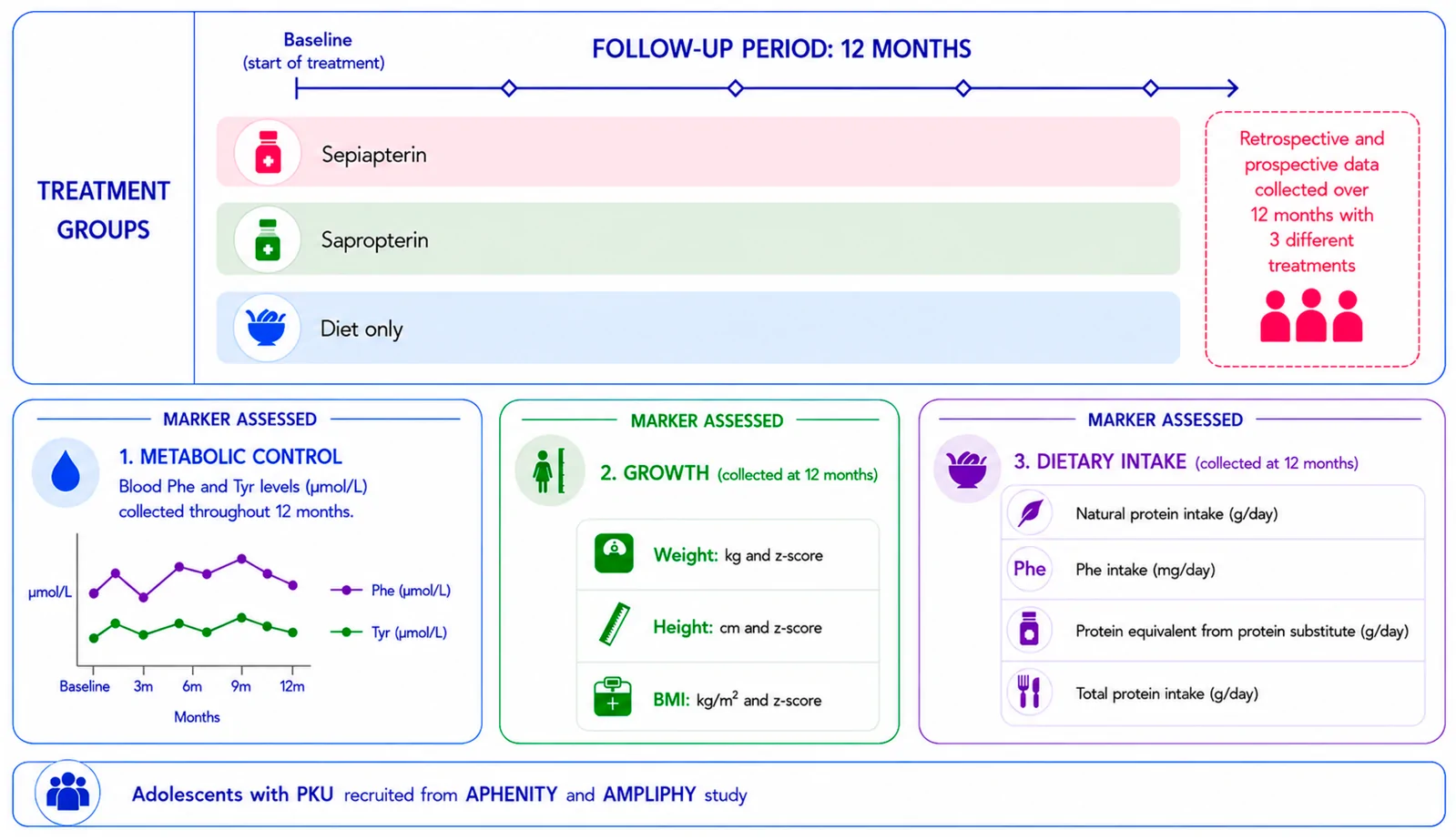
Study design.

Dietary data were collected at the end of each 12-month treatment period for dietary management alone, sapropterin (where applicable), and sepiapterin. The study design and timing of dietary assessments are illustrated in Figure 1.

Sepiapterin treatment data were collected between February 2023 and January 2026, and sapropterin treatment data between July 2023 and August 2024. Historical data for dietary management alone were collected between October 2015 and January 2024. Although most participants commenced sapropterin treatment in 2023, participant 3 had previously participated in sapropterin research trials; consequently, historical diet-only data for this participant extended back to 2015.

### 2.2. Inclusion Criteria

Eligible participants were early-treated adolescents with PKU who were diagnosed through newborn screening, were receiving follow-up at Birmingham Children’s Hospital, were aged 13–19 years and were receiving sepiapterin treatment. Exclusion criteria were late-treated PKU; primary tetrahydrobiopterin (BH4) deficiency due to pathogenic variants in GTP cyclohydrolase 1 (GCH1), 6-pyruvoyltetrahydropterin synthase (PTS), quinoid dihydropteridine reductase (QDPR), sepiapterin reductase (SPR), or pterin-4 apha-carbinolamine dehydratase 1 (PCBD1); and individuals not receiving sepiapterin treatment.

### 2.3. Ethical Permission

This study was approved in the UK by the London—Brighton & Sussex Research Ethics Committee with the reference 22/LO/0485 and the Integrated Research Application System (IRAS) number 1005744. The ‘Declaration of Helsinki’ (64th World Medical Association General Assembly, Fortaleza, Brazil, October 2013) and Good Clinical Practice guidelines were followed during the study.

### 2.4. Statistical Analysis

As this was a consecutive case review series including all eligible adolescents treated with sepiapterin at our centre, no formal sample size calculation was performed. The primary outcomes were blood Phe concentrations, natural protein intake, protein equivalent from protein substitutes, and total protein intake. Continuous variables are presented as mean ± standard deviation (SD) and median (range), while categorical variables are summarised as frequencies and percentages. Due to the repeated-measures nature of the dataset, blood Phe measurements were not considered independent observations. To avoid pseudoreplication, repeated Phe measurements were summarised at the individual patient level for each treatment period. Pairwise differences between treatment periods were assessed using the two-tailed Wilcoxon signed-rank test, restricted to patients with data available for both treatment periods. Because treatment exposure was not available for all patients across all periods, the number of paired observations varied between comparisons. *p*-values were adjusted for multiple comparisons using the Holm method. A two-sided *p*-value < 0.05 was considered statistically significant. Statistical analyses were performed using GraphPad Prism (version 10.1.0; GraphPad Software, Boston, MA, USA).

## 3. Results

### 3.1. Participants

Seven adolescents with early-treated PKU (five males and two females) were included in this retrospective case series. Median age at initiation of sepiapterin was 13 years (range, 12–17 years). Five participants had previously demonstrated responsiveness to sapropterin, while two were non-responsive and had remained on dietary management alone before commencing sepiapterin. All participants were identified through newborn screening. Median blood Phe concentration at diagnosis was 1329 μmol/L (range, 890–1610 μmol/L); the lowest diagnostic concentration occurred in an infant identified following sibling screening. Baseline demographic, clinical, and genotypic characteristics are presented in Table 1.

The five sapropterin-responsive participants had received sapropterin at the maximum licensed dose of 20 mg/kg/day before transitioning to sepiapterin. All seven participants received sepiapterin at the licensed dose of 60 mg/kg/day, administered as a single daily dose.

### 3.2. Metabolic Control

**Figure 2:**
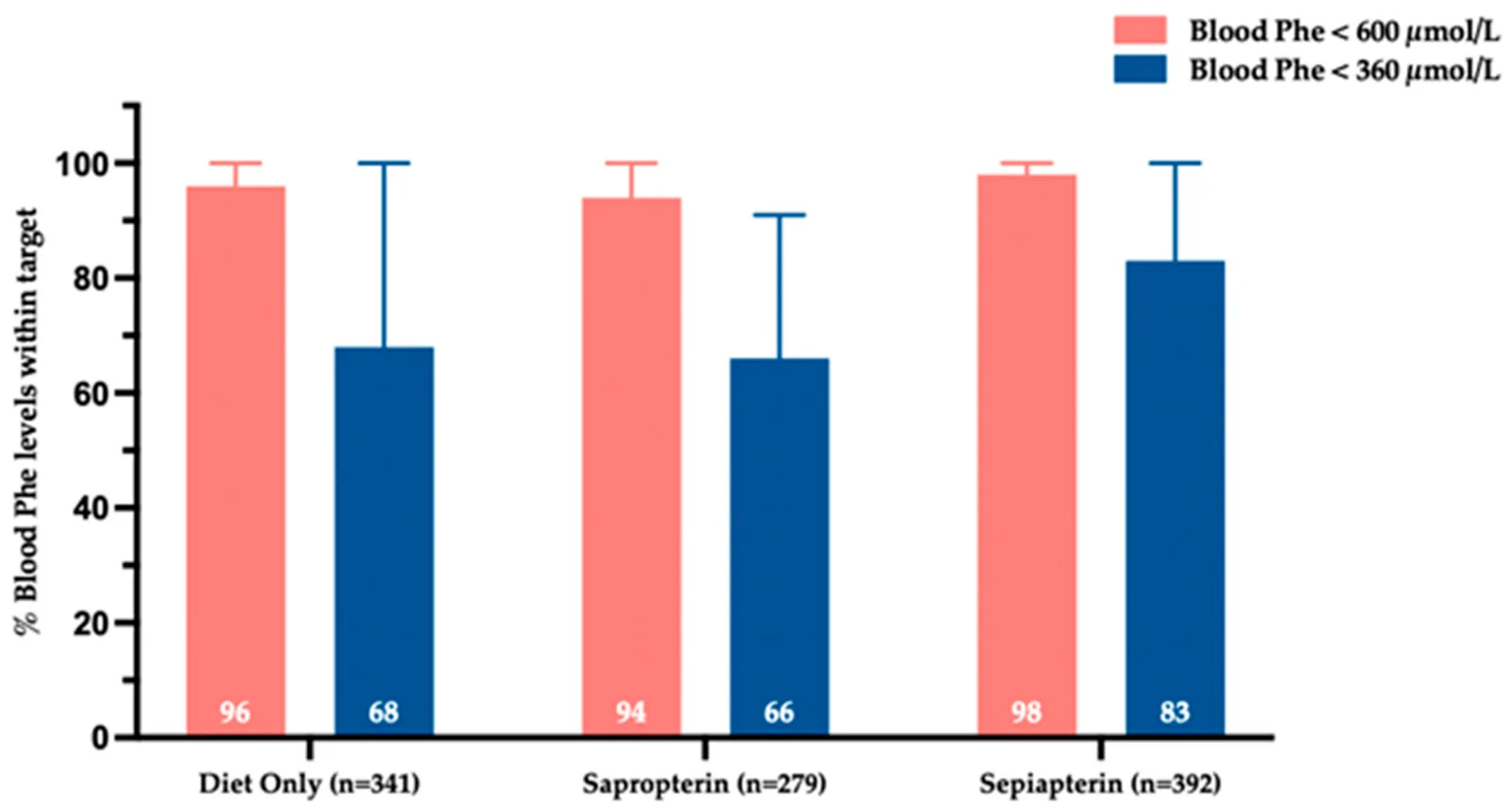
Percentage of blood Phe measurements within clinically relevant guidelines [5,30] according to treatment. Bars show for seven subjects the percentage of total blood phenylalanine measurements ≤ 360 µmol/L and ≤600 µmol/L during dietary treatment alone (n = 7), sapropterin treatment (n = 5) and sepiapterin treatment (n = 7). Error bars indicate the reported ranges. Numbers in parentheses represent the total number of blood spot measurements.

Mean (SD) blood Phe concentrations were lowest during sepiapterin treatment (260 ± 116 μmol/L) compared with diet alone (303 ± 157 μmol/L) and sapropterin treatment (321 ± 136 μmol/L), accompanied by reduced inter-individual variability (Table 2). Mean blood tyrosine concentrations remained similar across all treatment periods. The proportion of blood Phe measurements within the recommended therapeutic target (≤360 μmol/L) increased during sepiapterin treatment, with a median of 83% (range, 77–100%), compared with 68% (range, 17–100%) during dietary treatment alone and 66% (range, 40–91%) during sapropterin treatment (Figure 2). Similarly, the proportion of blood Phe measurements below 600 μmol/L remained consistently high across all treatment periods but was greatest during sepiapterin treatment (median 98%, range 93–100%) compared with dietary management alone (96%, range 72–100%) and sapropterin treatment (94%, range 83–100%). Blood Phe and tyrosine concentrations, together with the proportions of blood Phe measurements ≤ 360 μmol/L and ≤600 μmol/L (according to American and European guidelines, respectively), are summarised in Table 2 and Figure 2 [5,30]. Individual data are presented in Supplementary Table S1.

Wilcoxon signed-rank test to analyse differences. Abbreviations: Phe, Phenylalanine; Tyr, Tyrosine.

### 3.3. Dietary Intake

Sepiapterin was associated with a marked increase in natural protein tolerance. Median natural protein intake increased from 6.5 g/day (range: 4.5–15.0) during diet-alone treatment (n = 7) to 20.0 g/day (range: 10.0–25.0) with sapropterin (n = 5) and 70.0 g/day (range: 34.0–90.0) after 12 months of sepiapterin treatment (n = 7). This represented a median increase of 218% (range: 70–471) compared with sapropterin and 600% (range: 200–1900) compared with diet alone.

**Figure 3:**
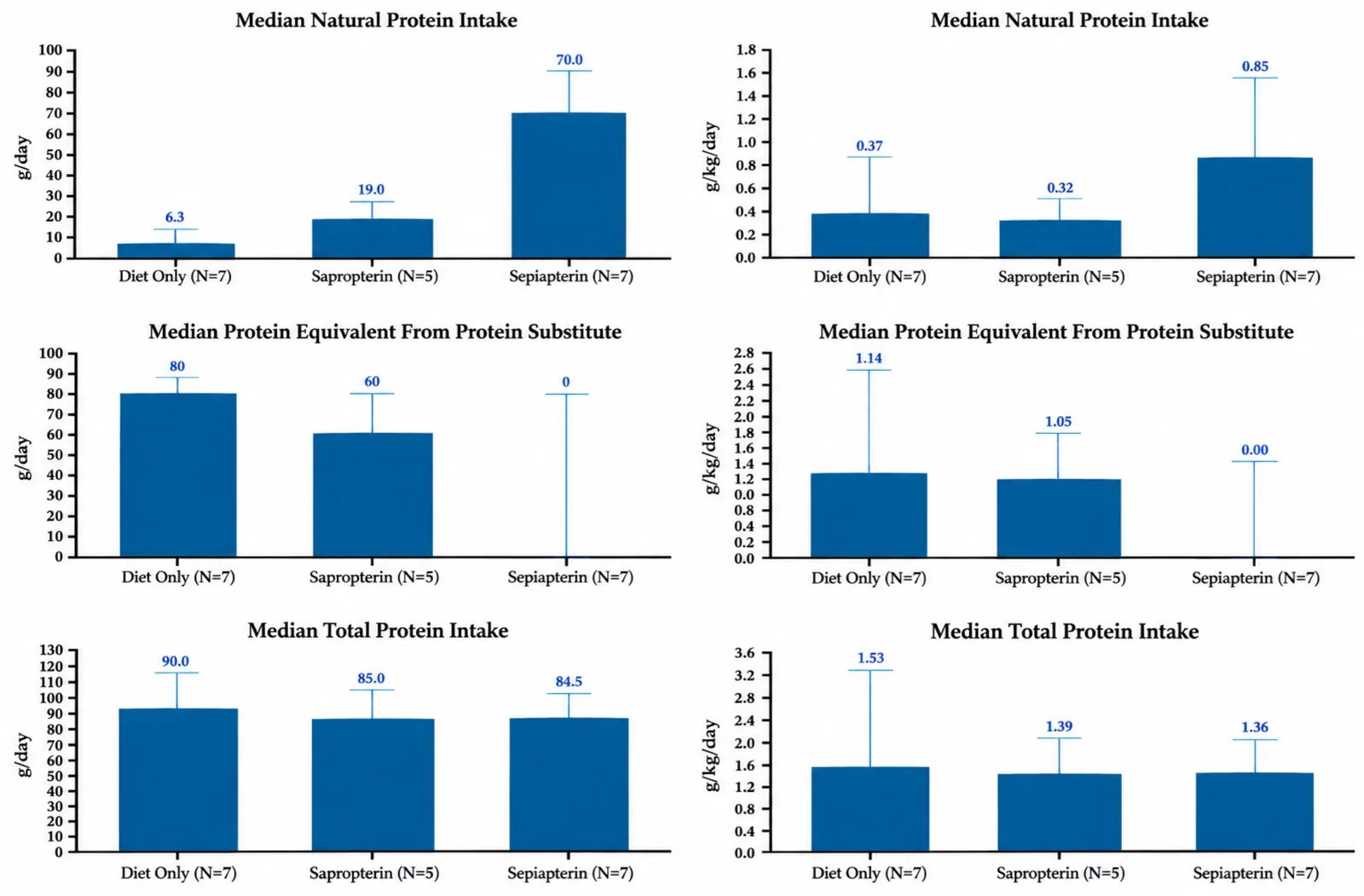
Protein intake (g/day and g/kg/day) during the study on three different treatments (diet only, sapropterin and sepiapterin) for seven subjects. Error bars indicate the reported ranges.

During sepiapterin treatment, median total protein intake was 90.0 g/day (range: 70.0–114.0), equivalent to 1.4 g/kg/day. Natural protein intake, protein equivalent from protein substitutes, and total protein intake across the three treatment periods are presented in Table 3 and Figure 3. Individual data are presented in Supplementary Table S2.

Sepiapterin treatment enabled substantial dietary liberalisation. All seven participants increased their natural protein intake, while four discontinued protein substitutes entirely. Of the remaining three participants, one maintained the same prescribed amount, one reduced their protein substitute intake, and one required an increased prescription to meet higher protein requirements associated with growth. Despite these changes, total protein intake remained nutritionally adequate throughout the study period (Figure 3).

Sepiapterin treatment facilitated the introduction of high-biological-value protein foods into the diet of all participants. All adolescents increased their natural protein intake, incorporating a wider range of protein-rich foods, including meat (7/7), fish (6/7), cheese (6/7), milk or yoghurt (5/7), and eggs (5/7). No participant reported any particular difficulties introducing these foods.

### 3.4. Monthly Dietary Protein Liberalization with Sepiapterin

**Figure 4:**
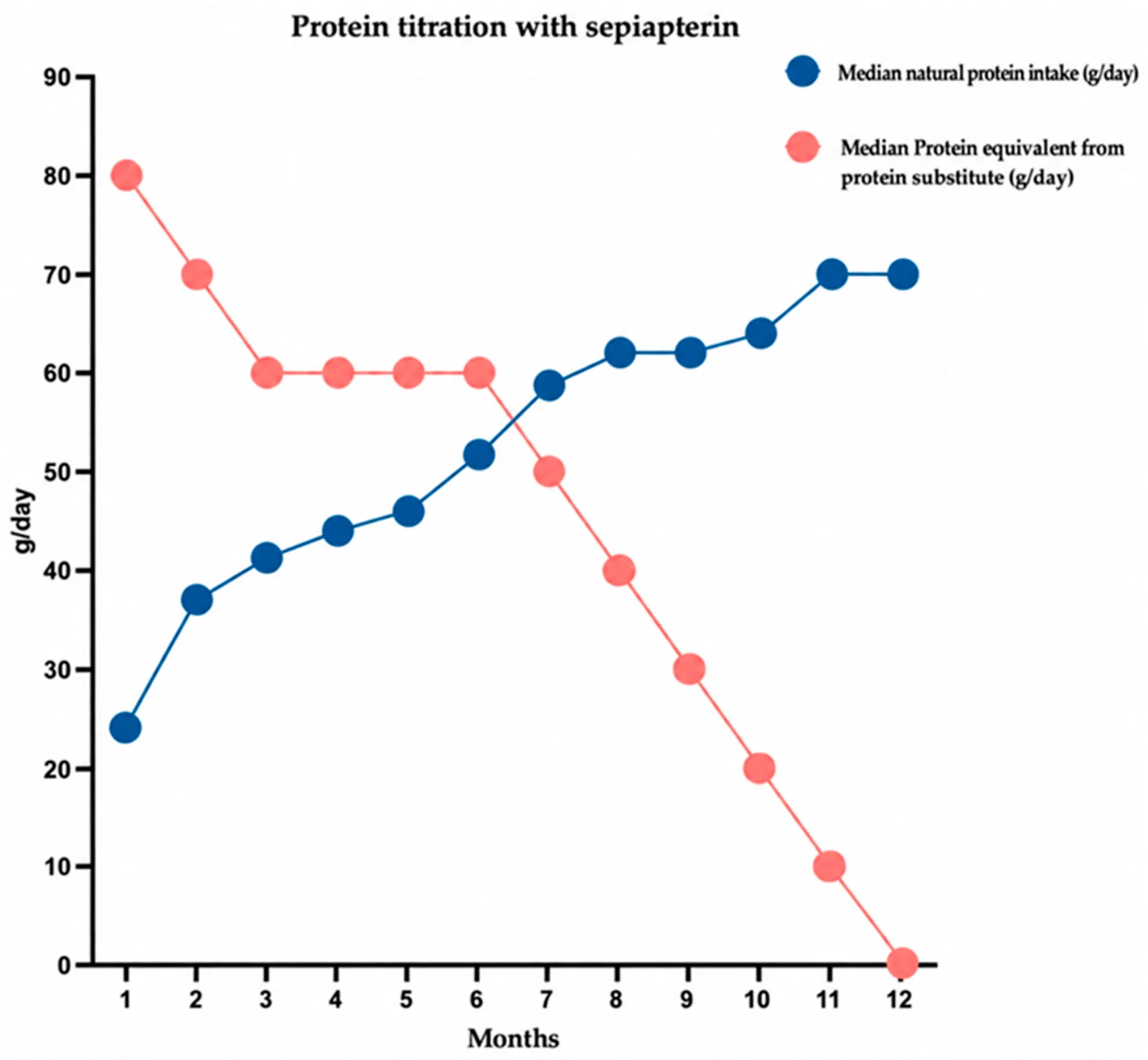
Median natural protein intake and protein equivalent from protein substitute by month with sepiapterin.

Sepiapterin treatment was associated with progressive dietary liberalisation over 12 months. Median natural protein intake increased almost threefold, from 24 g/day after 1 month to 70 g/day at 12 months (Figure 4), accompanied by a marked reduction in reliance on protein substitutes, with the median protein equivalent decreasing from 80 g/day at month 1 to 0 g/day at month 12. Four of the seven participants discontinued protein substitutes entirely, three within 8 months and the remaining participant by 12 months.

### 3.5. Growth

Weight and height z-scores increased over each 12-month study period, reflecting expected growth during adolescence (Table 4). Although linear growth was maintained, the prevalence of overweight and obesity also increased. At baseline, two participants were overweight and one was obese. Following 12 months of sepiapterin treatment, three participants were overweight and two were obese, with one participant moving from a healthy weight to overweight and another from overweight to obesity.

### 3.6. Adverse Events on Sepiapterin for 12 Months

No serious adverse events or treatment-related adverse events were observed during the 12-month sepiapterin treatment period. Reported intercurrent illnesses included two episodes of hay fever, one episode of a throat infection, one episode of infectious mononucleosis (glandular fever), and one upper respiratory tract infection requiring antibiotic treatment. All events resolved without discontinuation of therapy and were considered unrelated to sepiapterin.

## 4. Discussion

This retrospective case series demonstrates that 12 months of sepiapterin treatment was associated with marked dietary liberalisation while maintaining excellent metabolic control in adolescents with PKU. Natural protein tolerance increased substantially compared with both sapropterin and dietary treatment alone, with median individual increases of 218% and 600%, respectively, enabling four of seven participants to discontinue protein substitutes completely. These dietary changes were accompanied by sustained metabolic control, with a median of 83% of blood Phe concentrations remaining within the recommended therapeutic target (≤360 μmol/L). Collectively, these findings suggest that sepiapterin may substantially reduce dietary treatment burden during adolescence, a period in which maintaining long-term adherence remains particularly challenging.

Sapropterin has significantly advanced the treatment of BH4-responsive PKU; however, its clinical utility is limited by responsiveness in only a subset of individuals and by relatively modest improvements in natural protein tolerance in many responders [31,32,33,34,35,36]. Long-term follow-up studies have also shown that some patients discontinue treatment because the dietary benefits are insufficient to justify ongoing therapy. In contrast, adolescents in the present series experienced substantial dietary liberalisation following sepiapterin treatment, including two participants who had previously shown no clinically meaningful response to sapropterin. Although this study was not designed to directly compare the efficacy of sepiapterin and sapropterin, these observations are consistent with findings from the phase III APHENITY programme, in which sepiapterin was associated with substantial reductions in blood Phe concentrations, high response rates, and increased natural protein tolerance, including among participants with previous exposure to sapropterin [27,28]. The metabolic control observed in our cohort is also consistent with findings from the Phase 3 AMPLIPHY study, in which sepiapterin produced greater reductions in blood Phe concentrations than sapropterin [29]. Taken together, these findings suggest that sepiapterin may provide clinically meaningful benefits across a broader spectrum of individuals with PKU than has historically been achievable with first-generation BH4 therapy.

Adolescence is a particularly vulnerable period for individuals with PKU, during which metabolic control frequently deteriorates as increasing independence, changing dietary habits and food preferences, peer influences, and ongoing maturation of executive function may compromise adherence to treatment [37]. Large multicentre studies have consistently demonstrated a decline in the proportion of adolescents achieving recommended blood Phe targets with increasing age [38,39]. In the present study, participants maintained good metabolic control throughout 12 months of sepiapterin treatment despite substantial dietary liberalisation and despite being older than during the historical comparator periods. This is clinically noteworthy because maintaining metabolic control commonly becomes more difficult during adolescence. However, the diet-only, sapropterin and sepiapterin periods were non-contemporaneous and occurred at different ages and stages of growth. Changes in body weight, dietary requirements, and treatment adherence may therefore have influenced the observed differences. Consequently, this retrospective case series cannot determine the extent to which the differences in metabolic control can be attributed specifically to sepiapterin. The historical comparisons should instead be regarded as supportive real-world context for the observed treatment response.

Although neurocognitive and psychosocial outcomes were not evaluated, sustained metabolic control during adolescence has the potential to reduce exposure to elevated blood Phe concentrations during a critical period of brain maturation, which may confer long-term benefits for cognitive function, educational attainment, and mental health. Whether these biochemical improvements translate into measurable functional benefits requires confirmation in prospective longitudinal studies.

An important observation was the progressive nature of dietary liberalisation following sepiapterin initiation. Natural protein intake continued to increase throughout the 12-month follow-up, accompanied by a progressive reduction in reliance on protein substitutes, suggesting that the full dietary effects of treatment may extend beyond the initial response period. Four participants discontinued protein substitutes completely, while the remaining participants achieved substantial increases in natural protein intake. Participants were also able to incorporate a wider range of high-biological-value protein foods, including meat, fish, eggs and dairy products, without reported difficulty. Collectively, these changes indicate a clinically meaningful reduction in dietary restriction and reliance on protein substitutes. Given the small cohort, these dietary outcomes are most appropriately interpreted in terms of the magnitude and direction of change rather than statistical significance.

However, quality of life, treatment satisfaction and adherence were not formally assessed using validated patient-reported outcome measures. Although reduced reliance on protein substitutes and less restrictive dietary management may reduce treatment burden, particularly during adolescence, this potential benefit cannot be established from the present study. Future prospective studies should therefore incorporate validated measures of quality of life, treatment satisfaction and adherence to determine whether dietary liberalisation translates into meaningful patient-reported benefits. Optimisation of dietary management following sepiapterin initiation remains an active and evolving process requiring ongoing specialist metabolic dietetic support, with regular reassessment of natural protein tolerance, nutritional adequacy and protein-substitute requirements [40].

An increase in the prevalence of overweight and obesity was observed during the 12-month follow-up. Interpretation should be cautious given the small sample size and the expected changes in growth and body composition during adolescence, together with the well-recognised increase in overweight and obesity with age [41,42,43]. It is therefore not possible to determine whether the observed changes in BMI were associated with sepiapterin treatment, dietary liberalisation, normal adolescent development, or a combination of these factors. Nevertheless, substantial dietary liberalisation may alter food choices, energy intake and overall dietary quality. Adolescents who have followed a highly restricted low-Phe diet from early childhood may have limited experience selecting, preparing and consuming unrestricted higher-protein foods. Dietary liberalisation should therefore be accompanied by continued specialist dietetic support, with counselling focused not only on increasing natural protein intake but also on healthy food choices, energy balance, portion size and overall dietary quality. Practical education in food selection and preparation, including cooking skills where appropriate, may also be beneficial. Regular monitoring of weight, BMI and nutritional adequacy remains important as dietary restrictions are relaxed. Larger prospective studies incorporating detailed assessment of energy and macronutrient intake, dietary quality, body composition, physical activity and cardiometabolic health are required to determine the long-term nutritional consequences of dietary liberalisation following sepiapterin treatment [44,45].

Sepiapterin was well tolerated throughout the 12-month follow-up, with no serious treatment-related adverse events observed. Episodes of intercurrent illness were associated with transient increases in blood Phe concentrations, consistent with the recognised metabolic effects of catabolic stress in PKU. These observations emphasise that, despite the improved metabolic control achieved with sepiapterin, ongoing blood Phe monitoring and appropriate dietary management remain essential during periods of illness to maintain metabolic stability.

This study has several limitations. The retrospective design and small sample size limit statistical power and the generalisability of the findings. The three treatment periods were non-contemporaneous, and participants were assessed at different ages and stages of growth. Changes in dietary habits, increasing independence, treatment adherence, body weight and nutritional requirements during adolescence may therefore have confounded comparisons between treatment periods. The observational design precludes conclusions regarding causality. Pre-sepiapterin dietary data were obtained from clinical records, and dietary assessment methods differed between treatment periods, with 24-h dietary recalls used during diet-only and sapropterin treatment and 3-day dietary records used during sepiapterin follow-up. Detailed assessments of total energy intake, macronutrient composition, dietary quality, body composition and biochemical nutritional status werenot available. In addition, patient-reported outcomes, health-related quality of life, treatment satisfaction and adherence were not formally assessed, limiting evaluation of the broader psychosocial and functional consequences of dietary liberalisation. Despite these limitations, the longitudinal within-patient observations provide clinically relevant real-world information on metabolic control and dietary liberalisation during sepiapterin treatment in adolescents and complement findings from the Phase 3 clinical programmes.

## 5. Conclusions

In conclusion, sepiapterin treatment was associated with sustained metabolic control and substantial dietary liberalisation over 12 months in adolescents with PKU. The marked increase in natural protein tolerance and reduced dependence on protein substitutes demonstrate a substantial reduction in dietary treatment requirements and suggest the potential for a meaningful reduction in treatment burden during adolescence. Confirmation in larger prospective multicentre studies is now required to establish the long-term clinical, nutritional, neurocognitive, and psychosocial benefits of this therapeutic approach.

## Figures and Tables

**Table 1:** Study patient characteristics.

Patient Number	Sex	Age at Baseline	Ethnic Origin	Diagnostic Phe Level (μmol/L)	Variant	Variant	Sapropterin Responsiveness
1	Male	12	Mixed race	1590	c.782G > A p.(Arg261Gln) missense	c.1315 + 1G > A p.? splice	Yes
2	Male	12	Mixed race	1400	c.926C > T p.(Ala309Val) missense	c.1103A > G p.(Glu368Gly) missense	Yes
3	Male	13	White British	1430	c.194T > C p.Ile65Th missense	c.1315 + 1G > A p.? splice	Yes
4 *	Male	13	White British	890^+^	c1222C > T p.(Arg408Trp) missense	c1223G > A p.(Arg408Gln) missense	No
5	Female	14	Eastern European	1610	c.782G > A p.(Arg261Gln) missense	c.1222C > T p.(Arg408Trp) missense	Yes
6 *	Male	16	White British	1010	c1222C > T p.(Arg408Trp) missense	c1223G > A p.(Arg408Gln) missense	No
7	Female	17	White British	1374	c1042C > G p.(Leu348Val) missense	C1222C > T p.(Arg408Trp) missense	Yes

* Patients 4 and 6 are siblings. ^+^ Patient 4 is a sibling screen therefore his diagnostic level is 890 μmol/L. Abbreviations: Phe, Phenylalanine.

**Table 2:** Metabolic control of adolescents in the study.

Treatment (Number of Blood Spots)	Blood Phe Levels (µmol/L) Mean ± SD Median; Range	% of Levels ≤600 µmol/L	% of Levels ≤360 µmol/L	Blood Tyr Levels (µmol/L) Mean ± SD Median; Range
Diet only (n = 341)	303 ± 157 280 (20–940)	96% (range 72–100)	68% (range 17–100)	48 ± 18 40 (20–170)
Sapropterin (n = 279)	321 ± 136 300 (50–730)	94% (range 83–100)	66% (range 40–91)	54 ± 16 50 (5–150)
Sepiapterin (n = 392)	260 ± 116 270 (30–810)	98% (range 93–100)	83% (range 77–100)	56 ± 14 60 (20–130)
*p-value sapropterin* vs. *diet only*	*p = 0.6250*			
*p-value* *sepiapterin* vs. *diet only*	*p = 0.3215*			
*p-value* *sepiapterin* vs. *sapropterin*	*p = 0.1250*			

**Table 3:** Dietary intake during the three different treatments.

Treatment (Number of Patients)	Natural Protein Intake (g) Mean ± SD Median; Range	Natural Protein Intake (g/kg) Mean ± SD Median; Range	Protein Equivalent from Protein Substitute (g) Mean ± SD Median; Range	Protein Equivalent from Protein Substitute (g/kg) Mean ± SD Median; Range	Total Protein Intake (g) Mean ± SD Median; Range	Total Protein Intake (g/kg) Mean ± SD Median; Range
Diet only (n = 7)	8.9 ± 4.6 6.5 (4.5–15.0)	0.25 ± 0.27 0.17 (0.08–0.85)	68.9 ± 15.1 80.0 (42.0–80.0)	1.62 ± 0.59 1.14 (0.91–2.49)	77.7 ± 14.4 84.5 (57.0–95.0)	1.88 ± 0.78 1.53 (1.0–3.2)
Sapropterin (n = 5)	18.2 ± 6.1 20.0 (10.0–25.0)	0.30 ± 0.10 0.32 (0.15–0.41)	68.0 ± 11.0 60.0 (60.0–80.0)	1.15 ± 0.31 1.05 (0.88–1.67)	86.2 ± 12.4 85.0 (70.0–102.0)	1.45 ± 0.34 1.39 (1.0–2.0)
Sepiapterin (n = 7)	64.5 ± 23.7 70.0 (34.0–90.0)	1.0 ± 0.48 0.85 (0.52–1.57)	26.4 ± 34.5 0 (0–80)	0.37 ± 0.50 0 (0–1.13)	90.9 ± 14.3 90.0 (70.0–114.0)	1.37 ± 0.30 1.36 (0.86–1.76)

**Table 4:** Growth of patients on diet only, sapropterin and sepiapterin.

Treatment (Number of Patients)	Weight (kg) Mean ± SD Median; Range	Weight (z-Score) Mean ± SD Median; Range	Height (cm) Mean ± SD Median; Range	Height (z-Score) Mean ± SD Median; Range	BMI Mean ± SD Median; Range	BMI (z-Score) Mean ± SD Median; Range
Diet only (n = 7)	47.5 ± 18.2 56.6 (17.5–66.1)	0.90 ± 0.85 0.94 (−0.09–2.11)	149.3 ± 22.4 155.2 (104.7–168.4)	0.48 ± 0.59 0.21 (−0.31–1.32)	20.4 ± 4.2 20.1 (15.1–27.4)	0.83 ± 1.08 0.79 (−0.84–2.36)
Sapropterin (n = 5)	61.1 ± 8.7 65.0 (47.6–68.0)	1.36 ± 0.45 1.33 (0.75–1.98)	164.0 ± 7.5 165.7 (156.1–174.2)	0.95 ± 0.94 1.33 (−0.37–1.77)	22.9 ± 3.8 23.3 (17.3–28.0)	1.23 ± 1.06 1.73 (−0.26–2.29)
Sepiapterin (n = 7)	67.8 ± 11.1 65.7 (51.0–81.5)	1.25 ± 1.15 1.50 (−0.83–2.22)	168.7 ± 5.7 170.9 (158.3–175.5)	0.49 ± 0.97 0.69 (−0.78–1.97)	23.9 ± 4.3 24.2 (17.4–30.0)	1.21 ± 1.22 1.54 (−0.62–2.48)

## Data Availability

The original contributions presented in this study are included in the article/Supplementary Material. Further inquiries can be directed to the corresponding author.

## Supplementary Materials

The following supporting information can be downloaded at: https://www.mdpi.com/article/10.3390/nu18203376/s1, Table S1: Metabolic control on diet only, sapropterin and sepiapterin for all patients in the study; Table S2: Dietary intake on diet only, sapropterin and sepiapterin for all patients in the study.
