# Supplementary material for: Sepiapterin Enables Liberalisation of Natural Protein Intake While Improving Metabolic Control in Adolescents with Phenylketonuria
Source: Nutrients. 2026 Oct 15;18(20):3376. doi: 10.3390/nu18203376 (PMC13708635; doi:10.3390/nu18203376)
Supplement: Supplementary file 1 [file nutrients-18-03376-s001.zip › nutrients-4588565-supplementary.pdf]

Supplementary Table S1. Metabolic control on diet only, sapropterin and sepiapterin for all patients in the study.

Patient number	% Blood Phe level <600 µmol/L on diet only	% Blood Phe level <360 µmol/L on diet only	% Blood Phe level <600 µmol/L on sapropterin	% Blood Phe level <360 µmol/L sapropterin	% Blood Phe level <600 µmol/L on sepiapterin	% Blood Phe level <360 µmol/L sepiapterin
1	98	86	99	69	99	81
2	100	88	100	91	98	92
3	100	100	83	67	100	80
4	95	47	N/A	N/A	98	78
5	89	57	100	40	100	77
6	100	51	N/A	N/A	93	86
7	72	18	90	70	100	100

Abbreviations: N/A, not available.

Supplementary Table S2. Dietary intake on diet only, sapropterin and sepiapterin for all patients in the study.

Patient number	Natural protein diet only (g/day)	Protein equivalent from Protein substitute diet only (g/day)	Total protein Diet only (g/day)	Natural protein sapropterin (g/day)	Protein equivalent from Protein substitute sapropterin (g/day)	Total protein sapropterin (g/day)	Natural protein sepiapterin (g/day)	Protein equivalent from Protein substitute sepiapterin (g/day)	Total protein sepiapterin (g/day)
1	6.5	60	66.5	20	60	80	34	80	114
2	5.0	80	85.0	14	80	94	80	0	80
3	15	42	57.0	25	60	85	45	45	90
4	15	80	95.0	N/A	N/A	N/A	90	0	90
5	6	60	66.0	10	60	70	42.2	60	102.2
6	4.5	80	84.5	N/A	N/A	N/A	90	0	90
7	10	80	90.0	22	80	102	70	0	70

Abbreviations: N/A, not available.
